# Impact of maternal respiratory infections on low birth weight - a community based longitudinal study in an urban setting in Pakistan

**DOI:** 10.1186/s12884-017-1275-y

**Published:** 2017-04-11

**Authors:** Asad Ali, Umber Zaman, Sadia Mahmud, Gul-e-Shehwar Zahid, Momin Kazi, William A. Petri, Zulfiqar Bhutta, Anita Zaidi, Molly A. Hughes

**Affiliations:** 1 Department of Pediatrics and Child Health, The Aga Khan University, Karachi, 74800 Pakistan; 2 Division of Infectious Diseases, University of Virginia School of Medicine Carter-Harrison Building, Room 1704, 345 Crispell Drive, Charlottesville, VA 22908 USA

**Keywords:** Pregnancy, Respiratory illness, ARI, Newborn weight, Longitudinal observational study

## Abstract

**Background:**

The health of mothers and their newborns is intricately related. The weight of the infant at birth is a powerful predictor of infant growth and survival, and is considered to be partly dependent on maternal health and nutrition during pregnancy. We conducted a longitudinal study in an urban community within Karachi to determine maternal predictors of newborn birth weight.

**Methods:**

Four hundred pregnant women were enrolled in the study during the period 2011–2013. Data related to symptoms of acute respiratory illness (fever, cough, difficulty breathing, runny nose, sore throat, headache, chills, and myalgia/lethargy) in the pregnant women were collected weekly until delivery. Birth weight of the newborn was recorded within 14 days of delivery and the weight of <2.5 kg was classified as low birth weight (LBW).

**Results:**

A total of 9,853 symptom episodes were recorded of fever, cough, difficulty breathing, runny nose, sore throat, headache, chills, myalgias/lethargy in the enrolled pregnant women during the study. Out of 243 pregnant women whose newborns were weighed within 14 days of birth, LBW proportion was 21% (*n* = 53). On multivariate analysis, independent significant risk factors noted for delivering LBW babies were early pregnancy weight of < 57.5 kg [odds ratio adjusted (OR_adj_) = 5.1, 95% CI: (1.3, 19.9)] and gestational age [OR_adj_ = 0.3, 95% CI (0.2, 0.7) for every one week increase in gestational age]. Among mothers with high socioeconomic status (SES), every 50-unit increase in the number of episodes of respiratory illness/100 weeks of pregnancy had a trend of association with an increased risk of delivering LBW infants [OR_adj_ = 1.7, 95% CI: (1.0, 3.1)]. However, among mothers belonging to low SES, there was no association of the number of episodes of maternal respiratory illness during pregnancy with infants having LBW [OR_adj_ = 0.9, 95% CI: (0.5, 3.5)].

**Conclusions:**

While overall respiratory illnesses during pregnancy did not impact newborn weight in our study, we found this trend in the sub-group of mothers belonging to the higher SES. Whether this is because in mothers belonging to lower SES, the effects of respiratory illnesses were overshadowed by other risk factors associated with poverty need to be further studied.

## Background

An estimated 358,000 women die annually due to complications that develop during pregnancy and childbirth [1]. For every woman who dies, at least 20 more suffer from pregnancy related injuries, infections, diseases and disabilities, often with lifelong consequences [2]. The health of mothers and newborns is intricately related, so improving outcomes in either requires effective nutrition, infection control measures, and antenatal care.

Pregnant women are vulnerable to viral respiratory infections [3, 4] and are considered to be at high risk for influenza [5] and its complications [6–8]. Preventing influenza in mothers leads to increase in birth weights [9] and reduced infections in newborns [10, 11]. While the impact of influenza during pregnancy on newborns is now better understood [7, 8, 12, 13], the importance of other respiratory infections has not been studied. In order to improve maternal and neonatal outcomes and to develop appropriate preventive and treatment strategies, the association between common maternal illness and newborn’s health needs to be studied. Therefore, we conducted this study to identify the association between maternal respiratory illnesses and infant birth weight in an urban slum of Pakistan.

## Methods

This study was conducted under approval by institutional review boards at the Aga Khan University, Karachi, Pakistan and University of Virginia, Charlottesville, Virginia, USA. Informed consent was obtained from the adult participants and by a parent’s informed consent for newborns. The study was conducted during the period from July 2011 to June 2013 in Bilal Colony, which is an urban settlement within Karachi, Pakistan. The total population of Bilal Colony is 76,361. The total number of women of reproductive age (15–49 years) is 17,351, and the number of children less than five years old is 11,023 (2010 baseline survey). This site has an ongoing demographic surveillance system (DSS) where baseline census has been performed. Information on the following are conducted routinely every three months: number of events of pregnancy, births, and deaths; migration in or out of the community; and number of married women.

A longitudinal, observational cohort study was conducted in which women in their first trimester or early second trimester of pregnancy were randomly selected from the list of pregnant women of Bilal Colony (DSS). Informed consent was obtained, and participants were enrolled and subsequently followed once weekly for symptoms of respiratory or febrile illness until delivery. The study was designed to document the timing and sequence of respiratory symptom episodes and to identify any association of these symptoms in the pregnant women to the newborn’s weight.

The sample size calculated for pregnant women was approximately 300 based on an assumption of 5% influenza rates in adults, with a margin of error of 5.62% and 95% confidence level. The total number of pregnant women enrolled in the study was 400 in order to account for attrition due to noncompliance with the study protocol, withdrawal from the study, and movement of participants out of the area.

Illness in the pregnant women was detected either by once weekly household visits by trained study health care workers, or through the pregnant women contacting project personnel at the field office, an easy walk from all of the homes enrolled in the study. On each weekly visit, the participants were questioned regarding symptoms of respiratory illness, diarrhea, vomiting and fever in the last seven days. A brief standard questionnaire was administered either at home or in the clinic if a pregnant woman had developed signs or symptoms of acute respiratory illness (ARI). The clinic in the field office is staffed with an obstetrics/gynecology medical officer who provided free care to the study participants and their immediate family, with health care available at the government hospital when the clinic was closed. The field teams encouraged the study participants to visit the clinic for any symptom. Appropriate and standard health care was provided to all the study participants according to the local standards.

### Statistical methods

Descriptive statistics and inferential data analyses were conducted using SPSS 17.0 [14]. Mean and standard deviation were computed for quantitative and proportions for categorical variables.

The outcome variable, weight of the newborns, was measured within 0–14 days of birth. The mean age of infants when the birth weight was recorded was 4.9 days (SD 3.7 days). The weight of the mothers was measured at the mean gestational age of 12.7 weeks (SD 4.4 Weeks). The signs and symptoms of maternal respiratory illness during pregnancy were recorded as the number of episodes of fever, cough, difficulty breathing, runny nose, sore throat, headache, chills, and myalgias/lethargy throughout the follow-up period. The number of episodes of each of these signs and symptoms per 100 weeks of pregnancy were computed. A principle component analysis [15] of the eight respiratory illness signs and symptoms episodes per 100 weeks of pregnancy was conducted. The first principle component explains 38%, and the second 17%, of the total variance of the 8 items. The values of eigenvector of the first principle component indicate that all 8 items contribute to the total score for the first component, and no item dominates. Scores based on the first component correlate highly with the total number of episodes of signs and symptoms of respiratory illness per 100 weeks of pregnancy (*r* = 0.989). Hence, in order to quantify maternal respiratory illness during pregnancy, we used the total number of episodes of signs and symptoms of respiratory illness per 100 weeks of pregnancy.

Multivariable logistic regression analysis was conducted to identify factors associated independently with infant LBW; LBW was categorized as < 2.5 kg. We conducted univariable logistic regression analysis for each of the socio-demographic and clinical characteristics of the pregnant mothers compared to delivery of LBW newborns. The criteria for selecting a variable for inclusion in the multivariable model was a (likelihood ratio test) *p*-value < 0.25 from the univariable logistic regression analysis, and variables considered to be of biological importance. In the multivariable model, scale of all continuous predictor variables was examined using the quartile analysis method. Confounding effects of variables, with insignificant Wald p-values in the multivariable model on other variables, were assessed. Biologically meaningful interactions between variables in the model were assessed for statistical significance [16].

## Results

Among the 400 women initially enrolled in the study, 157 were lost to follow-up by the time of delivery, mainly due to migration before or at the time of delivery. Out of the 243 infant deliveries, 21% (*n* = 51) were LBW newborns. At the first clinic visit of the pregnant mother, the average age and average weight of the pregnant women in the study were 24.1 years and 56.1 kg, respectively. Thirty two percent (n=76) of the mothers had primary education or above, and only 26% (n=62) of the women had antenatal visits during their pregnancy. The mean age of mothers (23 years) was found to be lower in those who had newborns with lower birth weights compared to those who had newborns with birth weight > 2.5 kg (24.4 years). In our study cohort, the major source of drinking water was piped water in the households, which was more frequently available for pregnant women who had newborns weighing >2.5 kg (36.9%) compared to pregnant women who had LBW newborns (31.4%). Use of water bought from drums was a more frequent practice of mothers of infants with LBW (37.2%) compared to mothers of infants with birth weight > 2.5 kg (29.7%) (Table 1).

**Table 1:** Descriptive Analysis of the characteristics of pregnant women with low birth weight infants compared to those with normal birth weight infants

Pregnant women characteristics	Sample size (n)	Birth weight	
		<2.5 kg (n = 51)	> = 2.5 kg (*n* = 192)
Age of pregnant women Mean(SD); years	243	23.0 (3.8)	24.4 (4.5)
Pregnant women’s education; n (%)			
No formal schooling	237	35 (68.6)	126 (65.6)
Below 10^th^ grade		8 (15.7)	38 (19.8)
10^th^ grade and above		6 (11.8)	24 (12.5)
Weight at first visit of pregnant women mean(SD); kg	243	49.0 (9.0)	53.6 (11.7)
Height of pregnant women mean(SD); cm	243	153.1 (4.5)	152.6 (11.5)
Gravidity, Median (IQR)	240	3 (2)	3 (3)
Household characteristics			
No of people living in household, Mean(SD)	243	8.9 (5.5)	8.7 (5.5)
No of rooms in the household Mean(SD)	242	2.5 (1.5)	2.4 (1.4)
Total score of household assets, Mean(SD)	243	154.3 (141.9)	201.1 (179.0)
Main source of drinking water; n (%)	242		
Piped into the house		16 (31.4)	71 (36.9)
Bought from tankers		16 (31.4)	63 (32.8)
Bought from drums		19 (37.2)	57 (29.7)
Use of flush toilet facility; n (%)	237	0 (0)	2 (1.0)
Use of boiled water; n (%)	243	10 (19.6)	40 (20.8)
Antenatal characteristics			
Age at first pregnancy (years) Mean(SD)	243	18.8 (2.2)	18.9 (3.6)
Child gestational age in weeks Mean(SD)	241	36.6 (1.2)	36.7 (3.9)
Number of antenatal visits during current pregnancy; n (%)	238		
0		36 (70.6)	140 (72.9)
1		5 (9.8)	9 (4.7)
2		4 (7.8)	23 (11.9)
3 and above		5 (9.8)	16 (8.3)
Clinical characteristics		Mean (SD)	Mean (SD)
Fever**~**	243	7.2 (7.4)	6.8 (7.9)
Cough **~**	243	10.2 (9.4)	8.5 (7.3)
Difficulty breathing **~**	243	10.6 (10.9)	8.2 (7.7)
Runny nose **~**	243	10.7 (8.5)	10.9 (7.3)
Sore throat **~**	243	10.0 (9.1)	9.1 (8.1)
Headache **~**	243	18.7 (13.3)	20.4 (13.1)
Chills **~**	243	6.6 (6.1)	5.3 (6.2)
Myalgias/Lethargy	243	19.0 (9.8)	18.0 (9.0)
Diarrhea **~**	243	3.3 (4.3)	4.6 (6.8)
Vomiting **~**	243	6.2 (9.0)	6.2 (8.9)
Total number of Respiratory symptom episodes* **~**	243	102.9 (49.7)	96.1 (41.3)
Infant characteristics		Mean (SD)	Mean (SD)
Weight of the infant(kg)	243	2.1 (0.2)	3.0 (0.4)
Height of the infant (cm)	243	45.6 (2.5)	49.5 (3.2)
MUAC** of infant (cm)	241	8.4 (0.9)	9.8 (0.8)
OFC*** of infant(cm)	242	31.7 (1.3)	34.2 (1.4)

* Respiratory symptoms included eight symptoms (cough, difficulty breathing, runny nose, sore throat, headache, chills, and myalgia/lethargy)

~ Episodes per 100 weeks of pregnancy

**Mid upper arm circumference

***Occipitofrontal circumference

The results of univariate logistic regression analysis are reported in Table 2. The socio-demographic characteristics of pregnant women associated with infants with low birth weight included age, gravidity, and maternal weight at first visit. A higher total score for household assets correlated with a decreased risk of delivering LBW infant. Increased gestational age was associated with a significantly reduced risk of LBW (Table 2).

**Table 2:** Univariate Logistic Regression Analysis of the characteristics of pregnant women to identify factors associated with low birth weight of infants (*n* = 222)

Pregnant women characteristics		Crude OR	(95% CI)	*P*-value
Pregnant women age (years)	For every 5 year increase	0.7	(0.5 0.9)	0.032
Age at first pregnancy (years)	For every 5 year increase	0.9	(0.6 1.5)	0.796
Gravidity (total number of pregnancies)	For every 1 unit increase	0.8	(0.7 0.9)	0.031
Weight at first visit (kg)	For every 10 kg weight increase	0.6	(0.5 0.9)	0.006
Height of pregnant women (cm)	For every 10 cm increase	0.9	(0.7 1.3)	0.739
Pregnant women education	No education	1		0.805
	Below high school equivalent	0.8	(0.3 1.8)	
	High school equivalent and above	0.9	(0.3 2.4)	
Household characteristics				
No of people living in house hold	For every 1 unit increase	1.0	(0.9 1.1)	0.766
No of rooms in the household	For every 1 unit increase	1.0	(0.8 1.3)	0.738
Total score of household assets	For every 200 unit increase	0.7	(0.4 0.9)	0.073
Source of drinking water	Piped water	1		0.578
	Water bought from tankers	1.1	(0.5 2.4)	
	Water bought from drums	1.5	(0.7 3.1)	
Use of boiled water	Yes	0.9	(0.4 2.0)	0.847
	No	1.0	-	
Antenatal characteristics				
Child gestational age in weeks	For every 1 week increase	0.4	(0.2 0.8)	0.000
First pregnancy	No	1		0.798
	Yes	0.9	(0.4 2.1)	
Number of antenatal visits during current pregnancy	For every 1 visit increase	1.0	(0.8 1.3)	0.828
Delivery location of last child	No previous pregnancy	1	(0.4 2.6)	0.815
	Maternity home	1.0		
	Home	1.2	(0.5 2.9)	
Clinical characteristics				
Maternal fever^a^	For every 10 episode increase	1.1	(0.7 1.6)	0.698
Maternal diarrhea^a^	For every 5 episode increase	0.8	(0.6 1.1)	0.178
Maternal vomiting^a^	For every 10 episode increase	0.9	(0.7 1.4)	0.969
Total number of Respiratory symptom episodes^a^	For every 50 episodes increase	1.2	(0.8 1.8)	0.313

^a^Episodes per 100 weeks of pregnancy

Complete data sets were available for 222 women for the multivariable model. There were a total of 9,853 symptom episodes recorded of fever, cough, difficulty breathing, runny nose, sore throat, headache, chills, and myalgias/lethargy of the pregnant women during their pregnancy. In the multivariable model, factors associated with LBW were lower weight of mother at first visit, higher gravidity, shorter gestational age of newborn, higher household socio-economic status, higher number of episodes of respiratory symptoms, and higher number of fever episodes. There were two interactions in the model; one between number of episodes of respiratory symptoms and SES, and another between gravidity and number of fever episodes. After adjusting for other variables in the model infants born to mothers with weight at first visit of < 57.5 kg were at 5-fold risk for LBW relative to mothers with weight ≥ 57.5 kg [ORadj = 5.1, 95% CI: (1.3, 19.9)] (Table 3).

**Table 3:** Multivariable Logistic Regression Model^a^ to identify factors associated with infant low birth weight (*n* = 222)

Variables	Adj OR	(95% CI)
Mother’s weight at first visit		
<57.5 Kg	5.1	(1.3 19.9)
≥57.5 Kg	1	-
Child gestational age (weeks)	0.3^b^	(0.2 0.7)
Association between number of episodes of respiratory illness /100 weeks of pregnancy and SES^c^		
*High SES*	1.7^d^	(1.0 3.1)
number of episodes of respiratory illness /100 weeks of pregnancy *Low SES*		
number of episodes of respiratory illness /100 weeks of pregnancy	0.9^d^	(0.5 3.5)
Interaction between number of fever episodes/100 weeks of pregnancy and Gravidity number of fever episodes/100 weeks of pregnancy		
*Gravidity* ^*e*^ *= 2*		(0.3, 2.2)
≥11	0.8	-
<11	1.0	
*Gravidity =3*		(0.5, 3.0)
≥11	1.3	-
<11	1.0	
*Gravidity =5*		(1.1, 10.5)
≥11	3.4	-
<11	1.0	
*Gravidity =7*		(1.4, 57.6)
≥11	9.0	-
<11	1.0	
*Gravidity =9*		(1.7, 357.2)
≥11	24.2	-
<11	1.0	

^a^. Adjusted for mother's age and number of episodes of diarrhea/100 weeks of pregnancy

−2 Log-Likelihood = 187.701, Hosmer & Lemeshow goodness of fit test *p*-value = 0.566

^b^. Odds Ratio (and 95% CI) corresponds to every one week increase in gestational age

^c^. SES (Socio-Economic Status). Total score of assets <142 is labeled as "low" and ≥ 142 as "high" SES

^d^. Odds Ratio (and 95% CI) corresponds to every 50 unit increase in number of episodes of respiratory illness /100 weeks of pregnancy

^e^. For values of "Gravidity" minimum = 1, maximum =11*, 25th percentile* = 2, *50th percentile* = 3, *75th percentile* = 5

There is evidence for an interaction between number of episodes of maternal respiratory illness symptoms/100 weeks of pregnancy with the pregnant mother's SES. This implies that the effect of maternal respiratory illness symptom episodes during pregnancy on infant LBW outcome depends on a mother's SES. Among mothers with high SES, for every 50-unit increase in number of episodes of respiratory illness/100 weeks of pregnancy the risk of infant’s LBW increased 1.7 times [OR_adj_ = 1.7, 95% CI: (1.0, 3.1)]. However, among pregnant mothers belonging to lower SES there is no significant association of number of episodes of maternal respiratory illness during pregnancy with a subsequent newborn with LBW [OR_adj_ = 0.9, 95% CI: (0.5, 3.5)] (Table 3). In addition, there was an interaction between number of fever episodes/100 weeks of pregnancy and gravidity in the multivariable model. If gravidity is 3 or less, there is no significant impact of maternal fever episodes during pregnancy on whether a newborn is LBW or not. However, with increasing gravidity there is a significant and progressively increasing adverse association of the number of maternal fever episodes during pregnancy with a newborn having LBW. Infants born to mothers who had ≥ 11 fever episodes/100 weeks of pregnancy had more than 3 times higher risk when gravidity was 5 [OR_adj_ = 3.4, 95% CI:(1.1, 10.5)] and 9 times higher risk when gravidity was 7 [OR_adj_ = 9.0, 95%CI:(1.4, 57.6)] relative to infants born to mothers who had < 11 fever episodes/100 weeks of pregnancy (Table 3).

## Discussion

We found that low maternal body weight during early pregnancy and gestational age of a newborn are significant risk factors for infant LBW in our study. In the population of women with a high incidence of respiratory infections, we found that an effect of the number of episodes of maternal respiratory illness/100 weeks of pregnancy on infant LBW may depend on the pregnant mother's SES, with the impact of maternal respiratory illnesses on infant birth weight being greater on women belonging to high SES. One explanation for this observation is that amongst women from low SES, there are other factors including poor maternal nutritional status, low education and inaccessibility to appropriate water sources that influence the weight of the newborn adversely. As a result, the effect of maternal respiratory illnesses on low birth weight is blunted and a trend of association was not shown in that subset of mothers with lower SES.

This study explores an association between newborn birth weights with the maternal respiratory morbidities during pregnancy in the community settings. Other studies have explored associations of vaginal infections [17] and periodontal infections [18] in pregnancy with delivering LBW infants. The strength of our study lies in the observational cohort design, where we actively followed pregnant women every week for presence of fever and respiratory symptoms in the previous seven days. Through this study design, we were able to demonstrate an appropriate temporal sequence between exposure of pregnant women with fever and respiratory illnesses and the outcome of LBW newborns. In our study sample, we found 21% of delivered newborns had LBW, which is comparable to the Pakistan Demographic and Health Survey data (26% low birth weight infants) for the period 2006–2007 [19].

In univariate analysis, we observed an association between increased parity and a decreased risk of having a newborn with LBW. It is consistent with the finding that second and third newborns usually weigh more than the first newborn in a family [20]. Similar to other previously published studies, this study supports that low SES of the pregnant mother is associated with an increase in the risk of having a newborn with LBW [21].

There are several limitations of the current study which include small sample size, assessment of illness by report only and limited ability to identify severity of illness. Although we enrolled 400 pregnant women, a 100 more than the required sample size to account for the expected loss of follow-up in this long surveillance study, 39% dropout was still higher than expected. Major reason for this dropout was migration of pregnant women to native places around the peri partum period. Another limitation is that the birth weight was recorded within 0–14 days of birth (mean age 4.9 days) due to the fact that in this community, access to the mother and the newborn baby can be difficult in the immediate post-partum period. The gestational age was measured by taking the date of the last menstrual period from the mothers instead of by ultrasound documentation due to logistic constraints. Finally, we could not assess some interactions for significance in the final multivariable model due to sparse data (that led to zero cell counts and hence numerically unstable logistic regression model).

## Conclusion

While overall respiratory illnesses during pregnancy did not impact LBW in our study, we found this trend in the sub-group of mothers belonging to the higher SES. Whether this is because in mothers belonging to lower SES, the effects of respiratory illnesses were overshadowed by other risk factors associated with poverty need to be further studied.

## Abbreviations

ARI: Acute respiratory infections
CI: Confidence interval
DSS: Demographic surveillance system
LBW: Low birth weight
OR: Odds ratio
PCA: Principal component analysis
SD: Standard deviation
SES: Socio-economic status.
